# Effects of soy isoflavonoids (genistein and daidzein) on endometrial receptivity

**DOI:** 10.22038/ijbms.2020.48294.11089

**Published:** 2020-12

**Authors:** Erdem Toktay, Jale Selli, Muhammed Ali Gurbuz, Tugba Bal Tastan, Rustem Anıl Ugan, Harun Un, Zekai Halici

**Affiliations:** 1 Kafkas University, Faculty of Medicine, Department of Histology and Embryology, Kars; 2 Alanya Alaaddin Keykubat University, Faculty of Medicine, Department of Histology and Embryology, Antalya; 3 Ataturk University, Faculty of Medicine, Department of Histology and Embryology, Erzurum; 4 Ataturk University, Faculty of Medicine, Department of Histology and Embryology, Erzurum; 5 Ataturk University, Faculty of Pharmacy, Department of Pharmacology, Erzurum; 6 Agrı Ibrahim Cecen University, Faculty of Pharmacy, Department of biochemistry, Ağrı; 7 Ataturk University, Faculty of Medicine, Department of Pharmacology, Erzurum

**Keywords:** Daidzein, Endometrial Receptivity, Genistein, Rat, Soy Isoflavonoids

## Abstract

**Objective(s):**

This study aimed to examine the effects of genistein and daidzein on endometrial receptivity by histopathological, immunohistochemical, and biochemical techniques.

**Materials and Methods:**

In this study, 72 female Sprague-Dawley rats were randomly divided into 8 groups. The endometrial receptivity model was applied to identified groups. Experimental animals were given periorally 10 mg/kg and high 40 mg/kg doses of genistein and daidzein for 5 days by gavage. At the end of the experiment, uterine tissues were evaluated histopathologically, immunohistochemically, and biochemically.

**Results:**

When histopathological findings were examined, significant decreases in pinopod formation were observed in high dose genistein and daidzein groups. When compared with the endometrial receptivity group, immunohistochemical staining findings showed a significant decrease in the expression of integrin β3, integrin αvβ3, LIF, and HOXA10 and an increase in MUC 1 expression in the high dose of genistein and daidzein groups. In biochemical evaluations, it was determined that genistein and daidzein increased estrogen levels and decreased progesterone levels in a dose-dependent manner.

**Conclusion:**

Genistein and daidzein have a negative effect on endometrial receptivity. Therefore, individuals with a risk of infertility should pay attention to the consumption of genistein and daidzein.

## Introduction

Soybean has been one of the main nutrients consumed for thousands of years in Far Eastern countries. Nowadays, Soybean has gained importance among agricultural products and has been used in the production of more than 400 industrial products due high protein contents, carbohydrates, fats, and minerals (1). 

Soybeans contain a particularly large number of isoflavonoids which are known as soy proteins. Genistein and daidzein are the best known soy isoflavonoids (2). Besides antioxidant (3) and anti-helminthic (4) activities, the isoflavonoids have been also shown to interact with estrogen receptors (5). Similarly, daidzein has been shown that it can alter estrogen metabolism in clinical studies (6). Due to similarity to the structure of 17 β-estradiol, both genistein and daidzein can compete for binding with estrogen receptors (5). In this respect, they are also known as endocrine system disrupting chemicals (7). In females, genistein has been reported to alter folliculogenesis and steroidogenesis (8). In another study, isoflavonoids were found to disrupt the function of steroidogenesis synthesis enzymes (9). 

Unexplained infertility has an important place in infertility cases. The problem of endometrial receptivity (ER), which is associated with implantation failure, may be a cause of this type of infertility (10). ER, which takes place in the presence of low estrogen and high progesterone, is reciprocal interaction between blastocysts and uterus and is characterized by pinopod formation and synthesis of adhesive molecules. Studies have shown that synthesis anomalies of pinopod formation and adhesive molecules occurring during this period can directly affect pregnancy success (11). The causes of these anomalies are divers and especially may be based mainly on nutrition or chemical exposure (12). In the literature, changes in the hormonal mechanism of estrogen and progesterone have been found to alter ER (13). 

In brief, it is inconceivable that genistein and daidzein, herbal estrogens, do not have any effect on ER. Therefore, in this study we aimed to investigate the role of genistein and daidzein on ER and relationship with unexplained infertility by histopathological, immunohistochemical, and biochemical techniques.

## Materials and Methods

***Ethics statement and animals***

This study (2018/131) was approved by the Local Ethics Committee for Animal Experiments of Ataturk University. Seventy-two Sprague-Dawley female rats with an average weight of 180-200 g and 10-12 weeks of age were obtained from the Laboratory of Atatürk University experimental Research and Application Center. The rats were given enough (*ad libitum*) water and pellet feed during the experiment. They were housed at normal room temperature and humidity levels.

***Animal grouping and drug administration***

Seventy-two experimental animals were randomly divided into 8 groups (n=9). The experimental groups are as follows: Non-Mating (NM: Non-Mating healthy group), ER , ER+Sesame oil (ER+SE), ER+Estrogen (ER+EST), ER+Genistein 10 mg/kg (ER+GE10), ER+Genistein 40 mg/kg (ER+GE40), ER+Daidzein 10 mg/kg (ER+DZ10) and ER+ Daidzein 40 mg/kg (ER+DZ40).

For ER model, rats in the estrus stage were selected by taking vaginal smears and the two females were left to mate overnight, with one male. The next morning the male rats were removed and this day was counted as the first day of pregnancy. The formation of the vaginal plaque was checked and pregnancy status was confirmed (14). Rats were sacrificed on the fifth day, as earlier studies considered the ER stage in implantation on the fifth day (12). Finally, conceptus structures were observed in uterine tubes after sacrification. 

Genistein (10 mg/kg and 40 mg/kg), daidzein (10 mg/kg and 40 mg/kg), and estrogen (5 mg/kg) were dissolved in sesame oil and given orally by gavage for 5 days starting from the day of the mating.

All of the chemicals used in our experiments, genistein, and daidzein were purchased from Alpha Aesar, Germany. Estradiol valerate was obtained from Sigma, Germany. As a solvent sesame oil was bought from Balen sesame oil, Turkey.

***Surgical procedures***

At the end of the experiment, all animals were anesthetized through IP administration of a combination of 15 mg/kg xylazine and 100 mg/kg ketamine. A longitudinal incision (3 cm) was created in the midline area of the lower abdomen. The structure of uterine horns was observed and carefully removed. Collected tissues were stored at 10% formalin for histopathological and immunohistochemical analyses. Finally, the blood sample for biochemical examination was taken directly from the heart and stored at -80 ^°^C.

***Histolologic analyses***

Uterine tissues were rapidly fixed in 10% formalin solution for 48 hr. After fixation, all uterine samples for histological tissue processing were routinely performed as described previously (15). After tissue processing, 5 micrometers thick sections were taken from each paraffin block for histopathological and immunohistochemical examination. Uterine tissue slides were stained with hematoxylin and alcian blue staining.

Immunohistochemical staining was performed using the Ventana BENCHMARK GX automatic immunohistochemistry staining system (16). Monoclonal Primary antibodies (Integrin β3, Integrin avß3, LIF, and MUC1: Santa Cruz; HOXA10: Biorbyt) were purchased and used in a 1:100 dilution.

All preparations were photographed by a Nikon Eclipse e600 microscope with a computer-aided camera.

***Biochemical analyses***

Whole blood samples were transferred to a vacuum plastic gel tube and were centrifuged at 4000 rpm. At the end of the centrifuge process, the serum fluid was taken into 2 ml storage tubes and stored at -80 ^°^C until the moment of measurement. Estrogen and progesterone measurements were performed with a rat specific ELISA kit (Biotec).

***Statistical and Semi-quantitative analysis***

The data of our study were statistically evaluated using IBM 20.00 SPSS program. The groups were compared with Tukey *post-hoc* tests on the one-way ANOVA multiple comparison test.

The evaluation of the Tukey test was performed by comparing NM, ER, and ER+EST groups. The symbols * (star) for the NM group, # (diesis) for the ER group, and Δ (delta) for the ER+EST group were used. Accordingly, for example, in the evaluation according to the NM group, *P*<0.05 for*, *P*<0.001 for **, and *P*<0.0001 for *** were expressed. 

In our study semi-quantitative scoring was performed. For alcian blue staining findings, in order to make the evaluation more clear between the groups according to the amount of mucin secretion seen on the epithelial surface, scoring was performed. In this case, mucin is scored with – for no or very small amount, + for small amount, ++ for medium amount, and +++ for excessive amount (17). And for immunohistochemical staining findings, at least five areas were evaluated for each uterus slide and the average staining density score was taken into account. (18). According to the scoring, immune positivity was scored with – (0%) for little or no, + (0-30%) for mild, ++ (30-60%) for moderate, and + + + (60-100%) for severe (19).

## Results

***Histopathological findings***

*Hematoxylin and eosin staining findings*

**Figure 1:**
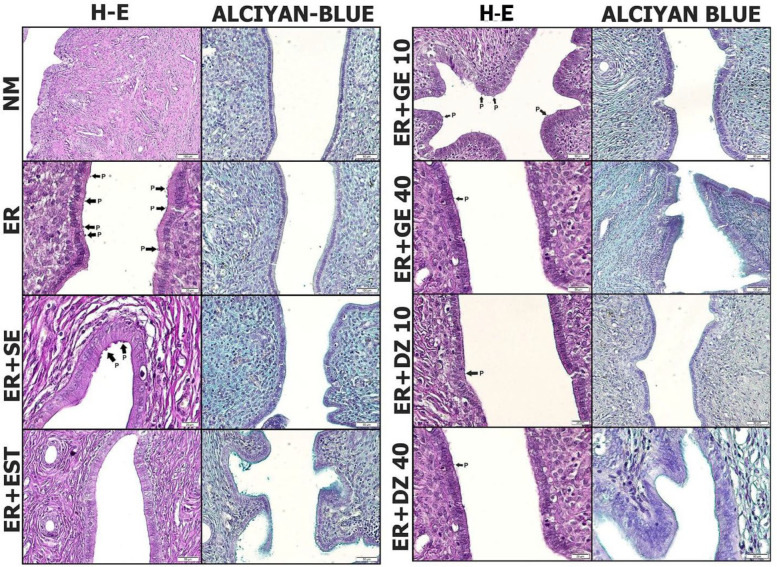
Hematoxylin-eosin stainingand alcian blue staining findings in uterus tissue

Histopathological examination of the images of the NM group showed that endometrium, myometrium, and perimetrium appeared to be healthy. Uterine glands and artery structures were seen in normal appearance in single-layer ciliated prismatic epithelium and lamina propria in the endometrium (Figure 1). In the ER group, pinopod formations were remarkable on the surface of the endometrium. In addition, enlarged uterine gland structures were observed (Figure 1). In the ER+SE group were seen pinopod formations and advanced uterine gland structures (Figure 1). In the ER+EST group, pinopod formations on the endometrium epithelium were very rare (Figure 1). In the ER+GE10 group pinopod formations showed in the lumen. However, fewer pinopod formations were seen compared with the ER group (Figure 1). In the ER+GE40 group, pinopod formations in lumen were rarely observed. It was also found that the gland structures were underdeveloped compared with the ER group (Figure 1). In the ER+DZ10 group pinopod formations showed in the lumen. However, fewer pinopod formations were seen compared with the ER group (Figure 1). ER+DZ40 group showed rare pinopod formations in lumen (Figure 1).

*Alcian blue staining findings*

According to alcian blue mucin staining results, amount of mucin was none or very little (-) in NM, ER, and ER+SE groups, less (+) in ER+GE10 and ER+DZ10 groups, medium (++) in ER+GE40 and ER+DZ40 groups, and excessive (+++) in ER+EST group (Table 1 and Figure 1).

*Immunohistochemical findings*

Semi-quantitative scoring was performed to understand the evaluation between the groups according to the density of immunopositive staining seen on the epithelial surface (Table 1).

**Figure 2:**
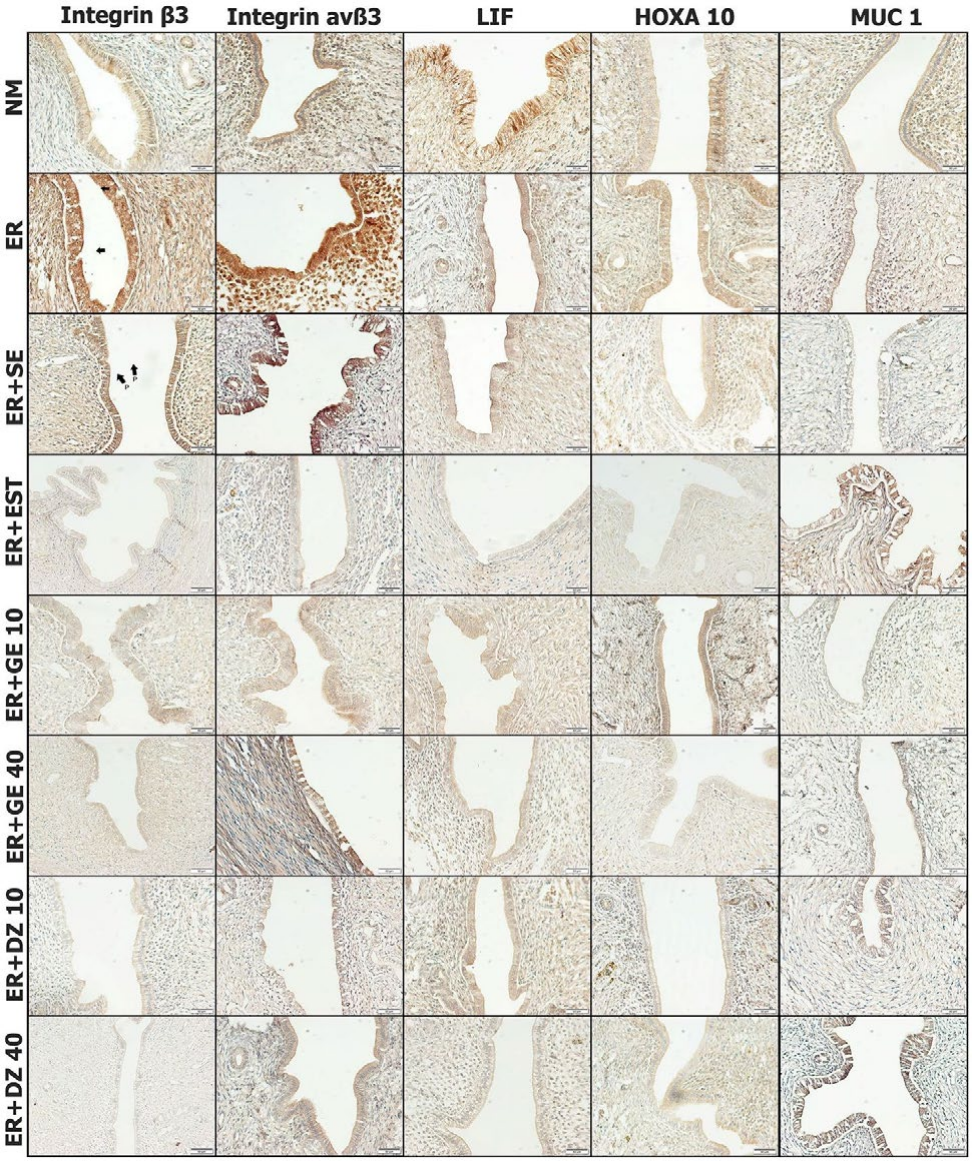
Integrin ß3, Integrin avß3, LIF, HOXA10 and MUC1 Immunohistochemical Staining findings in uterine tissue

Immunohistochemical staining with Integrin β3 antibody showed mild (+) immune positivity in NM, ER+GE40, ER+DZ10, and ER+DZ40 groups, moderate (++) in ER+GE40 group, severe (+++) in ER and ER+SE groups, while in ER+EST Group immune negativity (-) was observed (Table 1 and Figure 2).

Immunohistochemical staining with Integrin avß3 antibody showed mild (+) immune positivity in NM, ER+EST, ER+GE10, ER+GE40, ER+DZ10, and ER+DZ40 groups, while severe (+++) immune positivity was observed in ER and ER+SE groups (Table 1 and Figure 2).

immunohistochemical staining with LIF antibody showed mild (+) in ER+GE10, ER+GE40, and ER+DZ10 groups, severe (+++) in NM Group, and middle (++) in ER and ER+SE groups, while immune negativity (-) was observed in the ER+EST Group (Table 1 and Figure 2).

Immunohistochemical staining with the HOXA10 antibody showed moderate (+) immune positivity in ER+DZ10 and ER+DZ40 groups and moderate (++), while immune negativity (-) was observed in ER+EST and ER+GE40 groups (Table 1 and Figure 2).

Immunohistochemical staining with MUC1 antibody showed mild (+) immunity in ER, ER+SE, ER+GE10, and ER+DZ40 groups, moderate (++) in NM and ER+GE40 groups, and severe (+++) in ER+EST and ER+DZ40 groups (Table 1 and Figure 2).

*Biochemical Findings*

**Figure 3:**
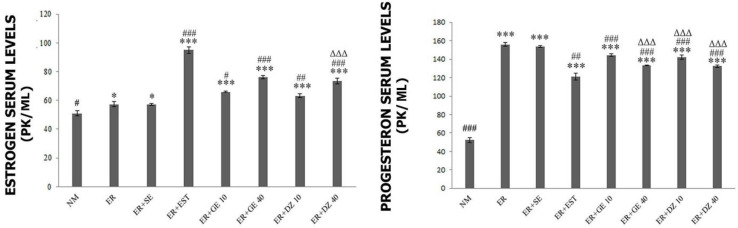
Serum levels of hormones estrogen and progesterone

Serum levels of estrogen according to intergroup assessment; In comparison with the NM group, ER and ER+SE groups showed significance (*), while ER+GE 10, ER+GE40, ER+DZ40, ER+DZ40, and ER+EST groups showed an advanced significance (***). In comparison with the ER group, there was significance (#) in NM and ER+DZ10 groups, moderate significance (##) in the ER+GE10 group, and advanced significance (###) in ER+GE40, ER+DZ40, and ER+EST groups. On the other hand, there was no significant difference between the ER and ER+SE groups. ER+GE40 and ER+DZ40 groups showed a significant degree of significance (ΔΔΔ) compared with the ER+EST Group (Figure 3). 

Serum levels of the hormone progesterone according to the intergroup assessment: in comparison to the NM group; ER and ER+SE, ER+GE10, ER+GE40, ER+DZ10, ER+DZ40, and ER+EST groups showed a high degree of significance (***). In comparison to the ER group, moderate significance (##) in ER+GE10, advanced significance in NM, ER+GE40, ER+DZ10, ER+DZ40, and ER+EST groups were observed. On the other hand, there was no significant difference between ER and ER+SE groups. ER+GE40, ER+DZ10, and ER+DZ40 groups showed a significant degree of significance (ΔΔΔ) when compared with the ER+EST Group (Figure 3).

P: Pinopod, NM: Non-Mating, ER: Endometrial Receptivity, SE: Susame Oil, EST: Estrogen, GE10: Genistein 10 mg/kg, GE40: Genistein 40 mg/kg, DZ10: Daidzein 10 mg/kg; DZ40: Daidzein 40 mg/kg Group- Magnification: x40

**Table 1:** Integrin ß3, Integrin avß3, LIF, HOXA10 and MUC1

Groups	İntegrin β3	İntegrin αvβ3	LIF	HOXA 10	MUC 1	Musin Amount
NM	+	+	+++	++	++	-
ER	+++	+++	++	++	+	-
ER+SE	+++	+++	++	++	+	-
ER+EST	-	+	-	-	+++	+++
ER+GE 10	++	+	+	++	+	+
ER+GE 40	+	+	+	-	++	++
ER+DZ 10	+	+	+	+	+	+
ER+DZ 40	+	+	-	+	+++	++

NM: Non-Mating, ER: Endometrial Receptivity, SE: Susame oil, EST: Estrogen, GE10: Genistein 10 mg/kg, GE40: Genistein 40 mg/kg, DZ10: Daidzein 10 mg/kg, DZ40: Daidzein 40 mg/kg Group- Magnification: x40

NM: Non-Mating, ER: Endometrial Receptivity, SE: Susame oil, EST: Estrogen, GE10: Genistein 10 mg/kg, GE40: Genistein 40 mg/kg, DZ10: Daidzein 10 mg/kg, DZ40: Daidzein 40 mg/kg- Magnification: x40

NM: Non-Mating, ER: Endometrial Receptivity, SE: Susame oil, EST: Estrogen, GE10: Genistein 10 mg/kg, GE40: Genistein 40 mg/kg, DZ10: Daidzein 10 mg/kg, DZ40: Daidzein 40 mg/kg Group, *: In comparison with the NM group, ER and ER+SE groups, #: In comparison with the ER group, Δ: In comparison with the ER+GE40 and ER+DZ40 groups

## Discussion

During ER, significant morphological, molecular, and biochemical changes occur in the uterus, especially in the endometrium. The presence or absence of these changes can be used as biomarkers for ER. Pinopods are the leading morphological markers related to ER. The routine menstrual cycle is 20-22 days, pinopod formations that occur between days play an important role in implantation. Because of this role, they are considered morphological markers (20). Studies have shown that many molecules associated with ER are associated with pinopods. In our study, we showed the presence of pinopods in ER and ER+SE groups associated with uterine receptivity in hematoxylin and eosin staining results. We also found that the increase in estrogen in the ER+EST group decreased pinopod formation. Indeed, Zhao and his colleagues had found similar findings to our findings in this study (21).

Many hormones and molecules have a role in the establishment of ER. In particular, there is a key association between low estrogen and high progesterone levels for ER. In fact, research has revealed that changes in estrogen and progesterone levels affect ER (22). It is suggested that daidzein and genistein may have an effect on ER. In our study, genistein and daidzein groups were observed to decrease the formation of pinopods depending on the dose in hematoxylin and eosin staining results. This conclusion explains the hypothesis that Zhenlong and colleagues in the study investigating the effects of daidzein on blastocyst implantation suggest that daidzein may be one of the factors that prevent implantation due to the deterioration in the hormonal axis related to the hypothalamus, pituitary, and gonads (23).

The biochemical findings of our study showed that the hormone estrogen has a negative effect on ER. The study by Zigler *et al.*, which describes the role of progesterone and estrogen in hormonal control of ER, supports our findings (24). In addition, we found that genistein and daidzein increase levels of the estrogen hormone depending on the dose and thus impair ER. In another study, which supports our study, isoflavonoids were shown to increase serum estrogen values in overectomized rats (25). 

In contrast to estrogen, progesterone is high during ER. In our study, it was observed that progesterone was high in ER and ER+SE groups, while it was observed that it showed low secretion in NM and ER+EST groups. This finding reveals that high estrogen plays a suppressive role against the hormone progesterone. In a study conducted by Ruihua and his colleagues, they found a decrease in serum progesterone levels in groups administered estrogen veratrine, as in our findings (26). We detected that these two isoflavonoids act as the estrogen hormone and reduce dose-dependent progesterone hormone levels as a suppressor. The findings of a study investigating the effects of low ovarian hormones on the prevention of breast cancer during the soy diet support our study (27).

The most common molecular biomarkers associated with ER are integrin β3 and integrin avß3, LIF, HOXA10, and MUC1. During the implantation window period, the adhesion molecules contribute to attachment of the embryo (28). The most known of these molecules are integrin molecules. Two members of this class, Integrin β3 and integrin avß3 adhesion molecules, have been preferred as supporting findings in studies related to ER (29-31). Research has shown that adhesion molecules have an important role in implantation failures (32).

In our study, we also used integrin avß3 and Integrin ß3, which are important biomarkers, to analyze the state of ER. Integrin ß3 was found to be highly expressed in ER and ER+SE groups in immunohistochemical findings. However, there was almost no expression in the ER+EST group. In addition, genistein and daidzein groups showed significantly reduced integrin β3 immune reactivity due to dose. As for immunohistochemical staining findings with integrin avß3, severe immune positivity was observed in the ER and ER+SE groups in parallel with our findings of integrin β3, while the severity of immunohistochemical staining was significantly reduced in ER+GE and ER+DZ groups depending on the dose. These two results show us that genistein and daidzein soy proteins lead to impairment by reducing the expression of integrin β3 and integrin avß3, especially when used at high doses.

LIF, a member of the interleukin 6 family, is one of the molecules required for embryo implantation (33). A study has suggested that abnormal LIF levels may be associated with infertility and low concentration of LIF levels may be associated with unexplained infertility (34). In our research, our immunohistochemical staining results showed that LIF synthesis decreased by daidzein and genistein administration dose-dependent. These results support anther study, which describes a potential autocrine/paracrine function in regulating embryo implantation of LIF and LIF receptor expression in the human endometrium (35). 

One of the molecules required for embryo implantation is the HOXA10 transcription factor. Research has revealed that HOXA10 is involved in inducing genes involved in the proliferation and differentiation of uterine stromal cells during the progesterone-dominant period (36). In relation to this situation, HOXA10 is an absolutely necessary factor in the embryo’s attachment period after fertilization. The immunohistochemical findings of our study revealed that the presence of the hormone estrogen in ER severely reduced the expression of HOXA10. In addition, genistein and daidzein soy proteins showed that dose-dependent HOXA10 led to reduced expression.

Mucins are glycoproteins with high molecular weight. Due to their high water retention capacity, they are secreted on the lumen surfaces of the digestive, respiratory, and reproductive organs especially to lubricate and moisturize the environment. Although more than 14 mucins are known in humans, they have been specifically associated with MUC1 endometrium (37). MUC1, located in the thick glycocalyx structure of the endometrium, has been reported to play a role in setting the correct location and time for the uterus in embryo implantation (38). MUC1 is also known as an antiadhesive molecule because of these properties. In our study, we showed that MUC1 immune positivity decreased in ER and ER+SE groups where pinopod presence was shown in our immunohistochemical findings. Furthermore, there were no mucin deposits in ER and ER+SE groups in alcian staining findings. In a study, in investigating presence of epitopes on the endometrial epithelium by scanning electron microscopy, MUC1 does not exist in pinopods which supports our findings (39).

Previous studies have shown that estrogen stimulates the hormone MUC1 expression, while high progesterone levels significantly decrease MUC1 expression (40). In our study, alcian staining results showed a large number of mucin deposits in the ER+EST group. While genistein and daidzein soy isoflavonoids groups were found to increase in dose-dependent mucin deposits in the endometrium. In addition to these findings, we observed that immunohistochemical findings significantly increased the expression of MUC1 in ER groups getting genistein and daidzein compared with the ER group.

## Conclusion

Unconscious and excessive consumption of genistein and daidzein may be a cause of unexplained cases of infertility due to impairment of ER. It has been suggested that there may be a relationship between increased soy production and cases of infertility in recent years. In short, individuals diagnosed with infertility or conditions that are considered risks for infertility should pay attention to soy consumption.
